# EGFR Targeting TKI-Related Skin Toxicities in a Patient with Darker Skin: A Case Report

**DOI:** 10.3390/curroncol29040205

**Published:** 2022-04-05

**Authors:** Arman Zereshkian, Alia Thawer, David M. Hwang, Susanna Cheng

**Affiliations:** 1 Department of Medicine, McMaster University, Hamilton, ON L8S 4L8, Canada; 2 Odette Cancer Centre, Sunnybrook Health Sciences Centre, Toronto, ON M4N 3M5, Canada; alia.thawer@sunnybrook.ca; 3 Department of Medical Oncology and Hematology, Sunnybrook Odette Cancer Centre, Sunnybrook Health Science Centre, Toronto, ON M4N 3M5, Canada; 4 Laboratory Medicine and Molecular Diagnostics, Sunnybrook Health Sciences Centre, University of Toronto, Toronto, ON M4N 3M5, Canada; david.hwang@sunnybrook.ca

**Keywords:** EGFR inhibitors, TKI, dark skin tone, lung cancer, skin toxicity

## Abstract

Epidermal growth factor receptor (EGFR) targeting tyrosine kinase inhibitors (TKIs) can result in significant skin toxicities that may impact patients’ quality of life. While these skin reactions are well documented in patients with lighter skin, there is a paucity of literature and images to guide clinicians in their assessment in patients with darker skin tones. Given that dermatological reactions in patients with darker skin are not well represented, this can result in the undertreatment or mistreatment of these otherwise common toxicities. Herein, we present a case of a female patient with a darker skin tone with metastatic non-small cell lung carcinoma (NSCLC) with EGFR-TKI-related skin toxicity and her clinical course.

## 1. Introduction

Challenges in detecting erythema and rash in patients with darker skin are well documented in the medical literature [1]. There is a lack of visual representation of various skin types in common teaching texts that can result in a delay in diagnosis or misdiagnosis in patients with darker skin [2]. In fact, a recent study in 2018 demonstrated that only 4.5% of images in medical textbooks represented darker skin tones [2]. Given the paucity of literature informing the clinical care of patients with a darker skin tone, this can have negative consequences for care, including missed diagnoses.

Epidermal Growth factor receptor (EGFR) targeting tyrosine kinase inhibitors (TKI) are commonly used as first-line treatment in patients with metastatic non-small cell lung carcinoma (NSCLC) who harbor a mutation in the EGFR protein [3]. These agents have shown superiority over standard platinum-based chemotherapy for the treatment of metastatic NSCLC in patients who harbor this mutation [4]. In fact, newer (third generation) EGFR-TKIs have demonstrated an improved overall survival as compared to first generation EGFR-TKI [4]. This oral treatment is less toxic as compared to standard chemotherapy, and given its improved survival, has become a standard of care as a first-line treatment in many jurisdictions.

The most common EGFR-TKI-related toxicities are dermatological in nature, including xerosis, pruritus, acneiform rash, and hair and nail changes [5]. The overall incidence of skin rash is reported to be between 47–100%, with up to 10% of these reactions being classified as severe (Grade 3/4) [5]. EGFR-TKI-related skin toxicity has been described as the most debilitating toxicity for patients receiving these oral therapies [6]. A recent observational study found that EGFR-TKI-related skin toxicity had a significant impact on the patient’s quality of life, with a poorer skin-related quality of life (SQROL) in those who had symptoms involving the scalp, face, and fingers [6]. If patients do not tolerate the treatment due to skin toxicities, they may prematurely stop treatment. Given the paucity of literature informing clinical care in patients with darker skin tones, there may be a failure to recognize and treat these very common side effects. This may be in part due to poor educational material, poor patient education, or clinician inexperience, which in turn may result in premature treatment discontinuation and/or increased morbidity and poorer SQROL.

Herein, we present a case of a patient with darker skin who presented with EGFR-TKI-related toxicity so as to guide clinicians in their approach to the diagnosis and treatment of these reactions.

## 2. Case Description and Clinical Course

A 56-year-old female, born in the Dominican Republic, with Fitzpatrick Type IV skin was diagnosed with Stage IVb non-small cell lung carcinoma (NSCLC) with metastatic disease to the lumbar and sacral vertebrae (L5-S1). Her past medical history was remarkable for hypertension and osteoarthritis, for which she had been on longstanding acetaminophen with codeine, lorazepam, and perindopril therapy. As a result of pain from metastatic disease in the spine, she was started on gabapentin therapy for neuropathic pain modulation. She also received palliative radiation for pain control to the lumbar and sacral vertebrae. Her biopsy results confirmed an EGFR positive mutation, and she was started on first line gefitinib 250 mg once daily in May 2019.

Within two weeks of starting gefitinib therapy, the patient developed a mild acneiform reaction on her face (Grade 1) for which she was prescribed topical corticosteroid therapy (hydrocortisone valerate 0.2% cream) twice daily, with modest symptomatic improvement. The rash was felt to be due to EGFR-TKI therapy, given that no other medications had recently been started and her other chronic medications (including her antihypertensive therapy) had been unchanged for several years. Her acneiform rash progressed with noted areas of hyperpigmentation on her lower back, and her topical therapy was switched to a combination of hydrocortisone valerate 0.2% cream and clindamycin 1% in a glaxal base cream to apply both to her face and lower back.

**Figure 1:**
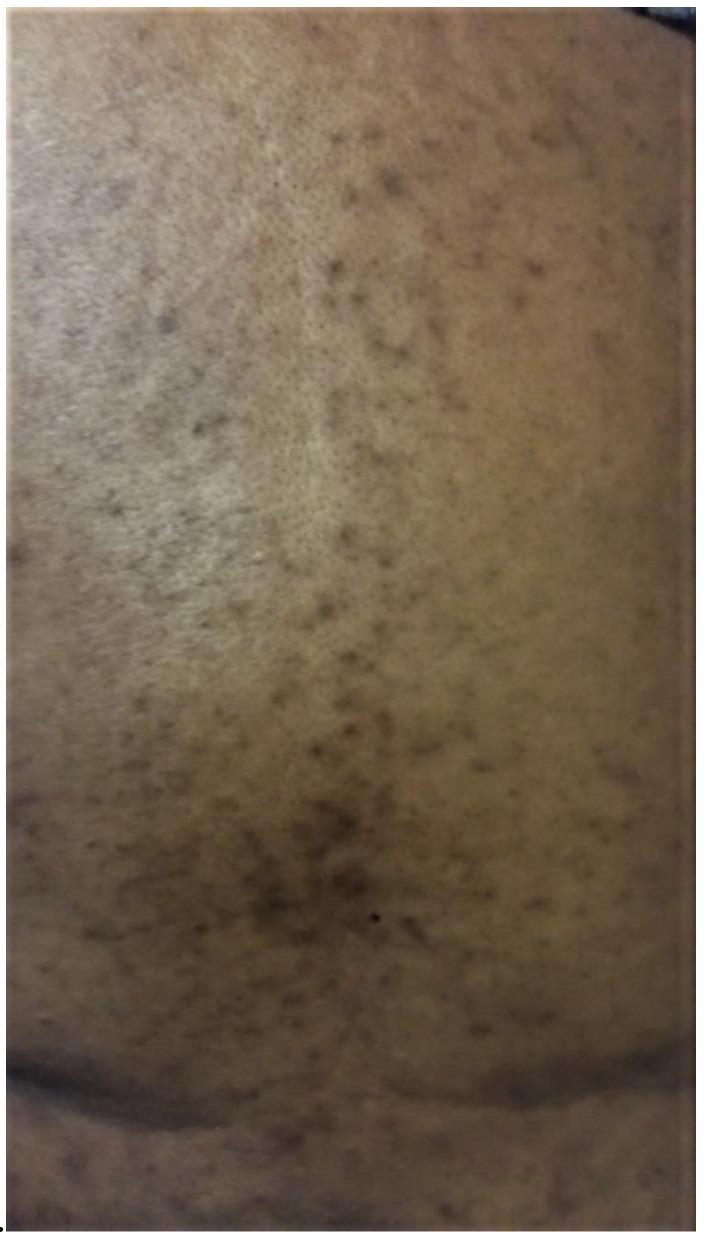
Hyperpigmentation of patient’s lower back with associated pinpoint scattered dark macules and papules along the spine. No active acneiform or papular eruptions are noted in this image. This picture was taken in March 2020.

**Figure 2:**
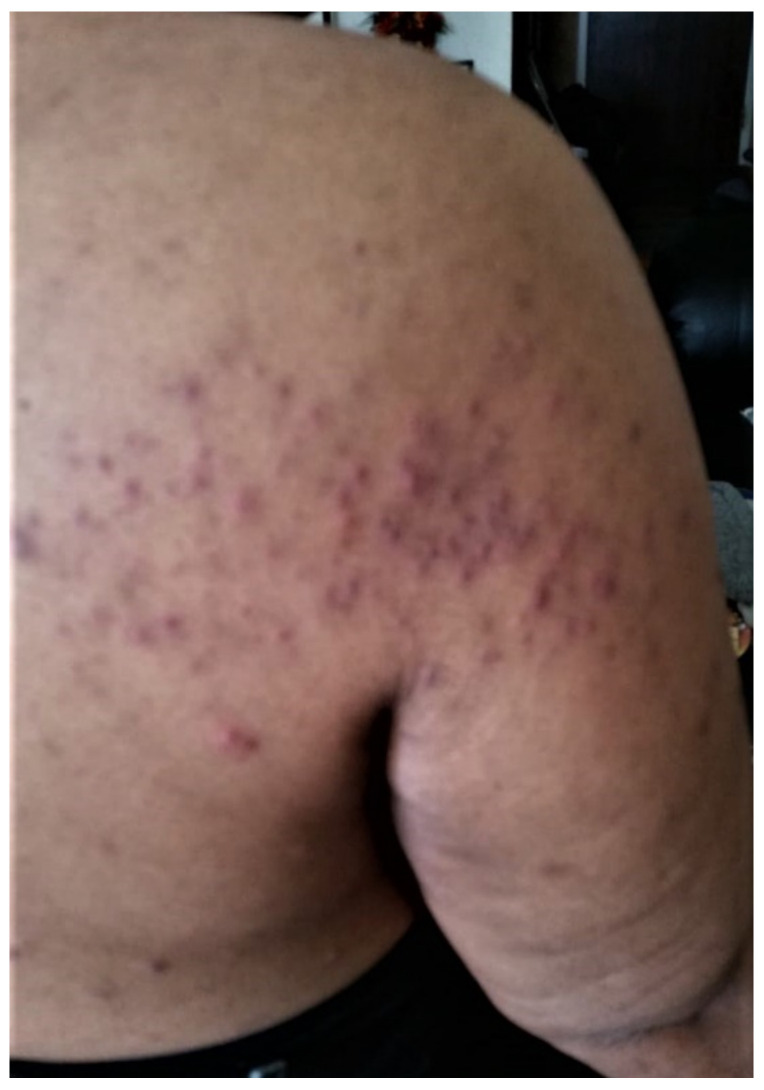
Scattered acneiform papular rash at various stages of eruption on an erythematous background centered along the upper back and lateral arms. A note should be made of the associated hyperpigmentation close to the axillary fold due to more maturing papular eruption. This photo was taken in April 2020.

The rash was considered “adequately controlled” by the patient, given the overall reduction in erythema; however, there were periods of increased papular erythematous acneiform rash noted along her upper back and arms over the next four to six months. Within 11 months of starting therapy, the patient presented to care with a worsening of the above-described rash (Figure 1 and Figure 2). The patient’s initial acneiform erythematous rash on her face had worsened, with faint areas of hyperpigmentation and hypopigmentation around the bridge of her nose. She was applying concealer and other make-up products rather than discussing this with her physician. Due to the COVID-19 pandemic, strained finances, and inadequate access to primary care, her supply of steroid and antibiotic cream was depleted by March 2020, and she discontinued these products. In April 2020, she was assessed through a virtual appointment (due to the COVID-19 pandemic) where significant xerosis, erythema was noted with worsening hyperpigmentation and hypopigmentation that had now involved the bridge of her nose and cheeks. Within one week of restarting her topical therapies, xerosis and erythema improved, but pigmentation changes remained.

**Figure 3:**
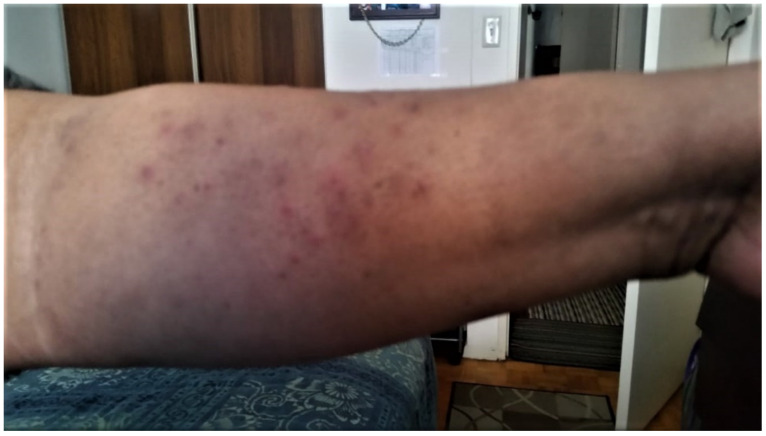
Xerosis and erythema of the arm with background hyperpigmentation. These areas of hyperpigmentation immediately distal to the elbow demonstrate resolving acneiform papular eruptions, while more distal to the area of hyperpigmentation, newer papular eruptions with more acute erythema are noted. This photo was taken in June 2020.

**Figure 4:**
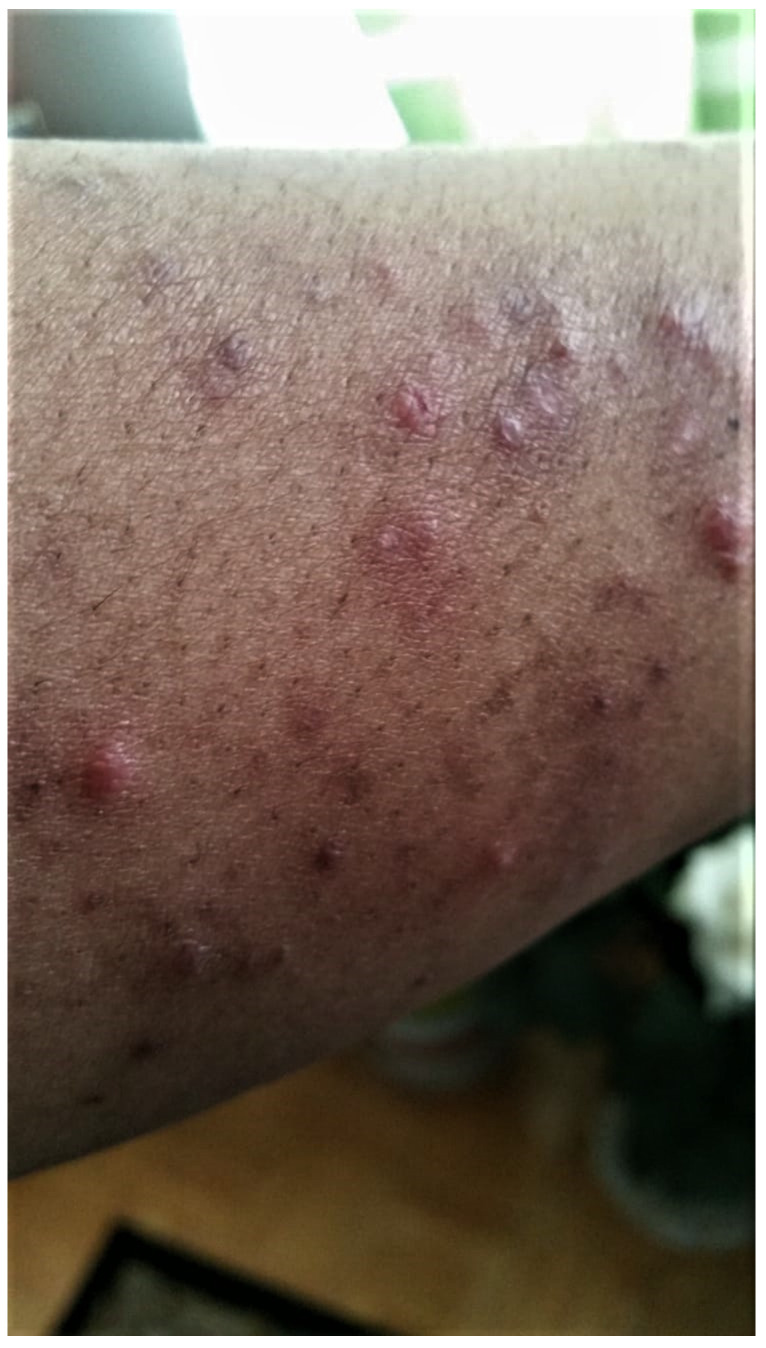
Acneiform eruption of papules along the arm of varying colors in different stages of inflammation. In the image, along the upper right portion of the arm, a coalescence of resolving papules can be seen on a background of very early pigmentation changes. This photo was taken in June 2020.

**Figure 5:**
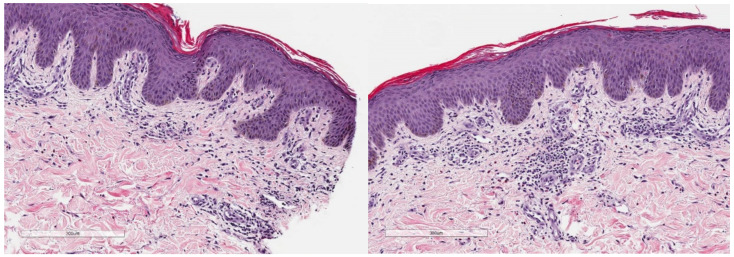
Hematoxylin and eosin stains of a skin biopsy of the patient’s intertriginous folds. The epidermis demonstrates focal parakeratosis, with the upper dermis demonstrating scattered mixed infiltrates of lymphocytes and eosinophils.

Over the course of the following month, even with continued topical therapy, the rash (Figure 3 and Figure 4) worsened with new pruritic maculopapular eruptions involving the skin along the mons pubis and intertriginous folds of the groin (with no fungal lesions). A severe skin reaction (Grade 3) was documented, and gefitinib therapy was discontinued. Oral doxycycline at 100 mg oral was initiated once daily. Unfortunately, the rash became more pruritic, and folliculitis was noted along the mons pubis region. Oral cephalexin therapy was prescribed for seven days three times daily, with no effect, prompting referral to a dermatologist. The dermatologist evaluation noted no fungal disease (with negative skin scraping on microscopy and culture). The skin biopsy of the groin demonstrated focal parakeratosis with mixed lymphocytic and eosinophilic infiltrates, overall compatible with dermatitis; however, whether these findings were related to gefitinib is unclear (Figure 5). Topical clindamycin was discontinued, and terbinafine cream was added twice daily to the above prescribed therapies (Hydrocortisone valerate, oral doxycycline, petrolatum jelly).

Given her persistent symptoms and previously documented intolerance to gefitinib therapy, having had this EGFR-TKI therapy held for a month, she was switched to another EGFR-TKI, osimertinib 80 mg once daily, after an exon-20 T790M mutation was identified. Over the next month, her rash slowly improved; however, there was noted hypopigmentation along the arms and legs, and the osimertinib dose was reduced to 80 mg every other day. Although no new erythematous eruptions were noted, areas of hypopigmentation and hyperpigmentation persisted.

## 3. Discussion

In this case, we present a patient with darker skin (FP IV) who presented with an ongoing complaint of xerosis, erythema, acneiform eruption and skin hypopigmentation and hyperpigmentation because of EGFR-TKI therapy, eventually resulting in the discontinuation of gefitinib due to a severe Grade 3 rash. In our review of the literature, only two case reports discuss EGFR monoclonal antibody-related toxicities in patients with darker skin [7,8]. There has been only one case report with a EGFR-TKI-related rash, which documented osimertinib-associated ashy dermatosis and hyperpigmentation (which the study’s authors felt was an unusual presentation) [9]. Given that EGFR-TKIs have been shown to cause a skin reaction in 47–100% of patients, it is likely that while clinicians come across patients with darker skin who are on EGFR TKI, these skin reactions are underreported and not documented well within the literature.

There are varying treatment recommendations around managing skin toxicities from EGFR-TKI therapies. In most patients, a preventative approach using fragrance-free emollients and daily cleansing is recommended with the addition of sun protection [10]. Thereafter, the approach varies; however, the mainstay of treatment includes topical antibiotics and steroids in addition to emollient therapy followed by oral antibiotics (such as doxycycline or minocycline), with the possible consideration of oral antihistamines for pruritis [10,11]. In patients who experience severe (grade 3 or 4) rash, a change in therapy is also warranted. In fact, a recent trial found that third generation TKIs (such as osimertinib) had fewer skin toxicities than traditional first-generation TKIs [12]. In our patient, a change from gefitinib to osimertinib was initiated for this reason.

Acneiform and papular erythematous rashes can result in skin hypo/hyperpigmentation. These sequelae are more pronounced in patients with darker skin [7]. The treatment of hypo/hyperpigmentation in this patient population is also significantly more difficult [7]. This reaction can also often occur with even mild to moderate acne, as was seen initially in our patient. This may be related to an increased melanosome response to inflammatory injury [7]. In our patient, the focus was predominantly placed on the acute acneiform reactions, but little comment was made around the patient’s skin hypo- and hyperpigmentation. As a result, therapy was not adjusted (early addition of oral doxycycline). Due to barriers around care (COVID-19 pandemic), the patient’s constrained finances, and poor patient education, the patient also discontinued her topical therapies, and her rash worsened with uncontrolled inflammation that contributed to worsening chronic pigmentation changes.

The delayed treatment of these skin pigmentation changes led to our patient using over-the-counter dermatological products to conceal these changes out of embarrassment. While not observed in our patient, a recent case report demonstrated a worsening exfoliating toxicity after a patient used over-the-counter lotions/products to treat EGFR-TKI for a mild acneiform rash [8]. Additionally, by delaying the initiation of oral doxycycline therapy, the patient’s rash worsened until it was deemed severe (Grade 3), and this eventually led to the discontinuation of the medication. Unfortunately, at this point, her pigmentation changes were chronic and remained unchanged after switching to osimertinib therapy. This underscores the importance of a prompt early treatment of drug rashes to avoid further sequelae. It also underscores the importance of an early referral to dermatologists, as they are more likely to appreciate hypo/hyperpigmentation changes and start early treatment. Given that EGFR skin toxicities are known to impact the quality of life, as was the case in our patient, early recognition and treatment could have improved the patient’s quality of life and prevented these chronic skin changes.

## 4. Conclusions

In conclusion, there is a paucity of data and case reports in the literature around EGFR-TKI-related skin toxicities in darker skinned patients. This paucity of data may result in an underappreciation of how these skin toxicities may manifest differently (higher rates of hypo/hyperpigmentation from even a mild acneiform reaction). This in turn can lead to undertreatment, a poorer overall quality of life, or premature treatment discontinuation/nonadherence. By reporting these adverse events with photos, we hope to aid clinicians in being mindful of their biases and in maintaining a low threshold of suspicion when treating EGFR-related skin toxicities in darker skinned patients, particularly given the possibility of chronic hypo/hyperpigmentation with delayed treatment.
